# Distinguishing complementary and alternative medicine: the role of religion, healthcare system satisfaction and country context

**DOI:** 10.3389/fsoc.2025.1702000

**Published:** 2026-01-16

**Authors:** Juliane Heise, Alexander Helbing, Peter Kriwy

**Affiliations:** Professorship of Sociology with a Focus on Health Research, Institute of Sociology, University of Technology Chemnitz, Chemnitz, Germany

**Keywords:** alternative medicine, CAM, complementary medicine, health-care system, religion

## Abstract

**Aim:**

This study aims to distinguish between the usage of complementary medicine and alternative medicine often jointly referred to as CAM. Furthermore, the analysis focuses on the role of religion, healthcare system satisfaction and the country of residence.

**Subject and methods:**

The analysis uses data of the International Social Survey Programme 2021 “Health and Health Care II” (ISSP 2021) to estimate the prevalence of complementary medicine and alternative medicine. A nested logistic regression model was applied to distinguish between no medicine use, conventional medicine, complementary medicine and alternative medicine.

**Results:**

The findings indicate that complementary medicine is significantly more prevalent than alternative medicine, though substantial cross-country differences are observed. While religious affiliation alone does not show a significant relationship with CAM usage, individuals who attend religious services regularly are more likely to use CAM in a complementary manner, alongside conventional medicine. Individuals who are dissatisfied with the health care system also are more likely to use both complementary medicine and alternative medicine. Higher levels of education are negatively associated with the use of alternative medicine. Younger individuals are more likely to use CAM and specific alternative medicine, compared to older age groups. Being female is consistently associated with a higher chance of CAM usage overall.

**Discussion:**

Treating complementary and alternative medicine as distinct reveals different prevalence rates and influencing factors. Religion, satisfaction with the healthcare system, education, age, and gender play varying roles depending on whether CAM is used alongside or instead of conventional medicine. Cross-country differences point to cultural and health system influences. For public health, distinguishing between complementary and alternative use can support more targeted strategies to promote safe integration and reduce risks from substituting conventional treatment.

## 1 Introduction

The use of complementary and alternative medicine (CAM; also called traditional or integrative medicine) has gained considerable attention in public health research over the past two decades (Ashrafizadeh and Rassouli, 2024; Bekke-Hansen et al., 2012; Bodeker and Kronenberg, 2002; Lee et al., 2022; Nicadao and Ai, 2014). Recent research reveals that on average 35 to 60% of adults in Europe and North America report engaging with CAM at some point in their lives (Alzahrani et al., 2021; Barnes et al., 2004; von Conrady and Bonney, 2017; Ernst, 2000). However, the term *complementary and alternative medicine* can be problematic. It combines two different health-related concepts: complementary medicine (a supplement to conventional medicine) and alternative medicine (a substitute to conventional medicine). Complementary medicine can be an attractive addition to conventional medicine. The placebo effect in particular has been proven to better individual health (Kaptchuk and Miller, 2015; Linde et al., 1997; Shang et al., 2005; Tavel, 2014). It works without side effects or invasive treatment, provided that no necessary medical treatment is neglected. The alternative use of CAM on the other hand presents a significant health concern. When sick individuals actively reject conventional medicine, they would normally need for healing, their health can be significantly harmed, potentially leading to higher morbidity and mortality rates. Therefore, a differentiated approach regarding the two kinds of CAM usage is essential.

There are two research gaps we want to address. Firstly, most of the existing studies investigate the prevalence and use of CAM but do not distinguish between the complementary medicine and alternative medicine (Alzahrani et al., 2021; Hanssen et al., 2005; Lee et al., 2022). Secondly many studies mainly use sociodemographic factors such as education and income to explain CAM usage (Bishop and Lewith, 2010; Frass et al., 2012). While these factors are indeed important, other potentially influential variables are neglected. We seek to address these research gaps through a separate analysis that distinguishes between the two forms of CAM, and by specifically focusing on religion and satisfaction with the healthcare system as explanatory variables. Religion profoundly shapes individuals’ worldviews, including their health-related decision-making (Chatters, 2000). Religious beliefs can influence trust in conventional medicine, openness to alternative therapies, and perceptions of health and illness. Some religious traditions emphasize holistic well-being, integrating physical, mental, and spiritual health, which aligns with many CAM practices (Koenig, 2012). In addition to belief related factors, satisfaction with the healthcare system represents another important determinant of CAM use. Lower satisfaction with the healthcare system is often associated with increased use of complementary and alternative medicine, as individuals may perceive these approaches as supplements or substitutes for conventional services, that are considered as inadequate (Tangkiatkumjai et al., 2020).

## 2 Theoretical background

### 2.1 Complementary medicine vs. alternative medicine

CAM can be defined as medical treatments that have no empirically proven effect beyond the placebo effect (Bausell, 2007). CAM also continues to be utilized even in the absence of demonstrable medical effects, whereas conventional empirical medical research and the scientific process normally leads to the discontinuation of methods that prove ineffective. This underscores another fundamental characteristic of CAM: it is typically not grounded in empirical and objective sciences such as biology, chemistry and human medicine. Instead, CAM often prioritizes a holistic approach, integrating physical, psychological, and spiritual aspects of health (WHO, 2024). Therefore, we define CAM as a diverse range of health treatments that are not based on empirical natural sciences and lacking empirical evidence (beyond the placebo effect) yet continue to be used to improve individual well-being. CAM includes a broad spectrum of therapeutic approaches, ranging from traditional medical systems such as Traditional Chinese Medicine, Ayurveda, and naturopathy to manipulative and body-based methods like chiropractic treatment and massage therapy (Nicadao and Ai, 2014). It also comprises biologically based approaches such as the use of herbal remedies, dietary supplements, and specific nutritional regimens, as well as mind–body practices like yoga, meditation, or tai chi, and energy-based therapies such as Reiki or therapeutic touch. These practices are typically aimed at improving subjective well-being, reducing stress, and fostering holistic health balance rather than directly treating biomedical diseases (National Center for Complementary and Integrative Health, 2021; Nicadao and Ai, 2014).

The term CAM combines complementary and alternative medicine (Čavojová and Ersoy, 2020). Complementary medicine refers to an individual utilizing both conventional medicine and CAM. In this context, “complementary” emphasizes coexistence and integration rather than substitution. Treatments are used alongside conventional medical care to support recovery, manage side effects, or enhance overall well-being. It is possible that the individual may lean more toward one form of treatment than the other. For instance, an individual might visit an acupuncturist every month but only sees a doctor once a year. Importantly, the use of complementary medicine does not imply a rejection of conventional medical care. Rather, it can be seen as an attempt to enhance personal well-being by adding treatments that go beyond standard biomedical care. Many individuals combine CAM with conventional medicine to relieve symptoms, improve quality of life, or maintain general health (National Center for Complementary and Integrative Health, 2021). Complementary medicine is often associated with a proactive health orientation, in which individuals seek to take more responsibility for their health without abandoning scientifically validated treatment. Typical examples include the use of relaxation techniques, acupuncture, or herbal supplements to complement ongoing medical therapy, such as pain management, cancer treatment, or chronic disease care. This type of usage may reflect dissatisfaction with certain aspects of conventional care, such as perceived lack of time or holistic attention, but not a fundamental distrust of biomedical science.

In contrast, alternative medicine refers to the exclusive use of such treatments while consciously rejecting conventional medicine. The use of alternative medicine can be problematic, especially when it involves serious or life-threatening conditions. Studies have shown that the replacement of conventional therapies with unproven alternative medicine methods can delay effective treatment and lead to poorer health outcomes (Horneber et al., 2012). While the desire for a healthy body and mind are universal, not all strategies to achieve this goal are equally effective. From an evidence-based perspective, conventional medicine remains the most reliable form of treatment for most acute and chronic conditions. It is therefore assumed that the exclusive use of CAM (meaning the use of alternative medicine) is relatively rare and limited to specific groups who, for ideological, experiential, or cultural reasons, reject conventional care (Berna et al., 2019; Frass et al., 2012). It is important to note that the distinction between conventional, complementary and alternative medicine is not always clear-cut and may vary across cultural and national contexts. In countries where traditional or indigenous healing systems are deeply integrated into the healthcare structure (e.g., China, India, or South Africa), such practices may not be perceived as “alternative” but as parallel or even primary forms of care. In contrast, in Western countries with predominantly biomedical health systems, CAM practices typically operate outside institutional medicine and are thus more clearly separated into complementary (used alongside) or alternative (used instead of) categories (Lee et al., 2022; WHO, 2024). These contextual differences should be considered when interpreting cross-national variations in CAM use. The distinction between complementary and alternative use is crucial when analyzing CAM behavior, as the motivations, outcomes, and implications differ significantly. Numerous studies have demonstrated that CAM is widely used across different populations, with 12-month prevalence rates ranging from around 24% to over 70%, depending on the country and study design (Horneber et al., 2012). On average, about one-third of the population reports using some form of CAM during a given year (Frass et al., 2012; Harris et al., 2012). Only a small number of studies have explicitly examined the difference between complementary and alternative medicine. Where this distinction has been made, findings suggest that complementary medicine is far more common, while alternative medicine remains a minority phenomenon (Astin, 1998; Berna et al., 2019). For example, Kemppainen and Kemppainen (2025) found out that approximately 8% of respondents rely on CAM without consulting medical professionals.

### 2.2 Factors explaining CAM usage

An important perspective expanding the understanding of health behaviors, including CAM usage, is the role of religion and spirituality. Religious beliefs and affiliations often shape individuals’ perspectives on health, illness, and healing (Koenig, 2012). Religion is conceptualized as an organized framework of beliefs, practices, and symbols aimed at fostering a connection with a higher power and typically involves a social dimension, including communal activities and commitments (Koenig, 2012). The concept of religious capital, introduced in sociological health research, provides a framework for understanding how religious resources like moral teachings, community support, and spiritual practices, affect health-related decisions (Oman, 2018). Some faith traditions emphasize holistic well-being, encouraging practices aligned with CAM philosophies, such as meditation, dietary restrictions, or herbal treatments (Oman, 2018). Empirical findings highlight complex associations between religious affiliation and CAM usage. For example, Bekke-Hansen et al. (2012) explored the relationship between religious faith and the use of complementary and alternative medicine for heart patients. Their findings suggest that faith plays a role in predicting CAM use following hospitalization. Studies have shown that individuals identifying with faiths such as Buddhism or New Age spirituality, which often integrate holistic health philosophies, are more likely to incorporate CAM into their healthcare strategies (Bekke-Hansen et al., 2012). In addition, religious beliefs can contribute to the substitutional use of CAM, leading some individuals to reject conventional medicine entirely. This can be due to a strong faith in divine or spiritual healing, skepticism toward scientific medicine, or the influence of religious communities that promote alternative healing practices over biomedical treatments (Barnes et al., 2004; Koenig, 2012). Overall religion therefore should promote usage of both complementary and alternative CAM usage.

*H1*: If a person is religious, this person is more likely to use complementary and alternative medicine.

From the perspective of individual cost–benefit calculations, people are more likely to turn to CAM when conventional care appears ineffective, overly costly, or insufficiently responsive to their needs. This reasoning can be directly linked to the broader healthcare system: when healthcare is perceived as expensive, impersonal, or of limited effectiveness, individuals may actively seek alternatives—this is where CAM becomes relevant. Satisfaction with the healthcare system has been identified as a key determinant of healthcare-seeking behavior, including the use of CAM (Fjær et al., 2020). Individuals who report high satisfaction with the accessibility, quality, and responsiveness of conventional services are more likely to consider these services sufficient for their health needs, thereby reducing the perceived necessity for complementary or alternative treatments. A frequently reported advantage of CAM is its emphasis on personal decision-making and individualized care, in contrast to the often rushed and standardized treatments characteristic of conventional medicine (Furnham, 2007).

*H2*: If a person is satisfied with the healthcare system of their country, this person is less likely to use complementary and alternative medicine.

Table 1 presents the estimated direction of effects for the use of CAM (complementary vs. alternative) compared to the sole use of conventional medicine.

**Table 1:** Estimated direction and strength of effect regarding the usage of CAM (complementary vs. alternative) compared to the sole use of conventional medicine.

Variables of the hypothesis	Complementary use	Alternative use
Religion	+	+
Satisfaction with the healthcare system	−	−

CAM = complementary and alternative medicine; + = positive effect; − = negative effect.

Although sociodemographic factors are not the primary focus of this study, previous research shows that gender, age and education consistently relate to CAM use and should therefore be accounted for. Women are generally more likely to use CAM than men (Abheiden, 2021; Fjær et al., 2020; Kemppainen and Kemppainen, 2025), which is often linked to stronger engagement in preventive care, self-management and holistic forms of treatment (Jones et al., 2019).

Findings on age are mixed: some studies report higher CAM use among older adults, partly due to chronic conditions, while others find greater use among younger individuals for wellness and lifestyle-related reasons (Bekke-Hansen et al., 2012; Wemrell et al., 2017). These inconsistencies highlight the importance of distinguishing complementary from alternative medicine, as motivations may differ by age.

Education shows an equally complex pattern. Higher education may reduce CAM use through greater critical appraisal of health information, but it may also increase complementary CAM use due to greater autonomy and scepticism toward conventional medicine (Astin, 1998; Moschèn et al., 2001). Recent work further suggests that individuals with higher education often possess higher levels of digital and critical health literacy, which facilitate distinguishing evidence-based information from misleading or promotional CAM claims circulating in digital environments (Del Arias López et al., 2023; van Kessel et al., 2022). Several studies indeed find that more educated individuals are more likely to use CAM, particularly as a complement to conventional care (Abheiden, 2021; Chan et al., 2012).

To account for these well-documented associations, gender, age and education are included as control variables in all models, while the main analytical focus remains on religion and satisfaction with the healthcare system.

## 3 Materials and methods

### 3.1 Study sample

The sample for this study is based on the International Social Survey Programme (ISSP) 2021 - “Health and Health Care II”^1^ dataset (ISSP Research Group, 2024). The ISSP conducts annual cross-national surveys on different topics relevant to social sciences. In 2021 the focus was on health and health care, providing a range of usable question items for the present analysis. The ISSP Research Group (2024) is implemented by national teams using probability samples, but target universes and coverage differ across countries with Mexico having the lowest (*n* = 1,001) and Switzerland having the highest (*n* = 3,349) sample size. Such differences affect statistical precision of country-level estimates (especially for low-prevalence outcomes like exclusive alternative medicine use) and may contribute to unequal stability of cross-national contrasts. We therefore interpret country differences with attention to these coverage variations. The complete list of countries and their corresponding sample sizes can be found in Supplementary Table 1. While the general target population is adults aged 18+, several countries fielded broader or capped age ranges (e.g., Austria, the Netherlands, and South Africa from age 16; Denmark 18–80; Finland 15–74; Norway 18–79), which can shift age compositions and affect comparability of prevalence estimates. Also, fieldwork periods (February 2021–April 2024) and data-collection practices differ by country. For example, Germany fielded the survey as a mail, self-administered questionnaire; the Netherlands implemented the module via the LISS probability panel; and South Africa conducted interviews face-to-face through regional fieldwork companies. The study documentation further provides a country-by-country summary of administration modes, indicating that some teams relied on single-mode implementations while others employed mixed-mode designs. These differences can influence response tendencies (e.g., willingness to self-disclose, item nonresponse) and should be considered when interpreting cross-national contrasts. In total *N* = 44.549 respondents from 30 countries participated in the survey. The respondents are, on average middle-aged (*M* = 49.46 years, SD = 17.46 years). A slight majority of respondents identify as female (53.80%), while 46.20% identify as male. A descriptive overview of the sample composition is available in Supplementary Tables 2.1, 2.2.

### 3.2 Operationalization

The dependent variable, the use of complementary and alternative medicine, is measured combining two questions. Respondents are asked: “During the past 12 months, how often did you visit or were visited by.?” - With the first question referring to “a doctor,” and the second to “an [alternative/ traditional/folk] health care practitioner.” Respondents who had no contact with either a doctor or a CAM practitioner were assigned to the group of no medicine use at all. Those who only consulted doctors and had no interaction with CAM practitioners are classified as users of conventional medicine only. Respondents who consulted both conventional doctors and CAM practitioners are categorized as users of complementary medicine. Finally, respondents who exclusively visited or were visited by CAM practitioners, are defined as users of alternative medicine. See Table 2 for the coding scheme of CAM usage. The definitional difference of alternative medicine and complementary medicine is therefore based on the difference in the number of visits of CAM practitioners compared to visits to a doctor.

**Table 2:** Coding of CAM usage using two variables (Q17a and Q17b) from the ISSP Research Group 2024 “Health and Healthcare II” dataset (with absolute number of respondents).

During the past 12 months, how often have you been visited by							
		… a CAM practitioner?					
		Never	Seldom	Sometimes	Often	Very often	Total
… a doctor?	Never	9.125	620	387	123	37	10,292
	Seldom	10.353	2.908	942	281	94	14.578
	Sometimes	8.284	1.775	2.073	399	89	12,620
	Often	2,855	682	559	484	84	4,664
	Very often	734	162	149	94	121	12,601
	Total	31,351	6,147	4,110	1,381	425	43,414

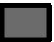
 No medicine; 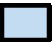 Conventional medicine; 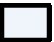 Complementary medicine; 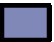 Alternative medicine.

For measuring religion, respondents are asked which religious main groups they belong to. Due to high multicollinearity (VIF = 173.49) between this variable (denominational belonging) and country of residence, it is recoded as a binary dummy variable (1 = no religion, 2 = religion). Denominational differences were initially considered but had to be collapsed into a binary indicator (religious vs. non-religious) due to high multicollinearity with country of residence. However, religious affiliation alone is not a precise measure of actual religious belief or practice, as individuals may formally identify with a religion without actively believing in or practicing it. Therefore, a second variable is included to better capture religious involvement. Respondents are asked how often they attend religious events or services. Their responses were grouped into five categories: (1 = never, 2 = less frequently than once a year, 3 = at least one time a year, 4 = at least one time a month, 5 = at least one time a week). No further measures of religiosity (e.g., belief intensity, private prayer, ritualistic practices) were available in the ISSP Research Group (2024) dataset. We therefore relied on the mentioned two indicators: formal affiliation and service attendance capturing both the organizational and participatory dimensions of religiosity.

Satisfaction with the healthcare system is measured using one question. The respondents are asked to rate how satisfied they are with the healthcare system of their country (“In general, how satisfied or dissatisfied are you with the health care system in [country]?”). Their responses are grouped into three categories: (1 = Neither satisfied nor dissatisfied, 2 = completely satisfied, very satisfied and fairly satisfied, 3 = fairly dissatisfied, very dissatisfied and completely dissatisfied). Because of the cross-sectional study design control variables are needed to handle possible biases. Therefore, sociodemographic variables are used. Gender is operationalized as a binary variable (1 = male, 2 = female) and age is kept as a metric variable based on the birth year. In the ISSP Research Group (2024) education is measured using the simplified International Standard Classification of Education (ISCED) 11. It will be further simplified in three categories (1 = no formal education and primary education, 2 = upper secondary and post-secondary education, 3 = short-cycle tertiary, lower-level tertiary, upper-level tertiary, PhD, and post tertiary specialization). Also, subjective health status is included as a control variable. Since CAM usage in this study is defined through visits to health care practitioners, there is a potential for bias among individuals in good health. For example, a person in good health might regularly use healing stones. Because their health is stable, the use of conventional medicine is not encouraged. However, if the same person were to fall seriously ill, they might also turn to a medical doctor. This pattern suggests that the use of CAM among healthy individuals may reflect well-being rather than treatment-seeking behavior, which could distort results. To address this potential bias, subjective health status will be assessed using the following question: “In general, would you say your health is …” The answer categories are (1 = fair and poor, 2 = good, 3 = excellent and very good). A detailed description of the whole coding scheme is presented in Supplementary Table 3.

### 3.3 Methods

The first step is a descriptive analysis of the dependent variable, with a particular focus on differences between countries. In the next step, a nested multiple logistic regression is applied to examine the relationship between CAM usage and the independent variables. This approach is chosen because the primary aim is to compare complementary and alternative medicine at the individual level, rather than to explain differences between countries. Since the dataset covers 30 countries, country dummies are included to account for national context without shifting the focus away from the main research questions. Missing data rates across the variables ranged from 0.00% (country) to 3.16% (religious attendance), resulting in an overall missing rate of 1.55%. Given this low proportion and in line with established methodological recommendations, listwise deletion was applied (Newman, 2014; Schlomer et al., 2010). Detailed missing data rates for all variables can be found in Supplementary Table 4.

**Figure 1:**
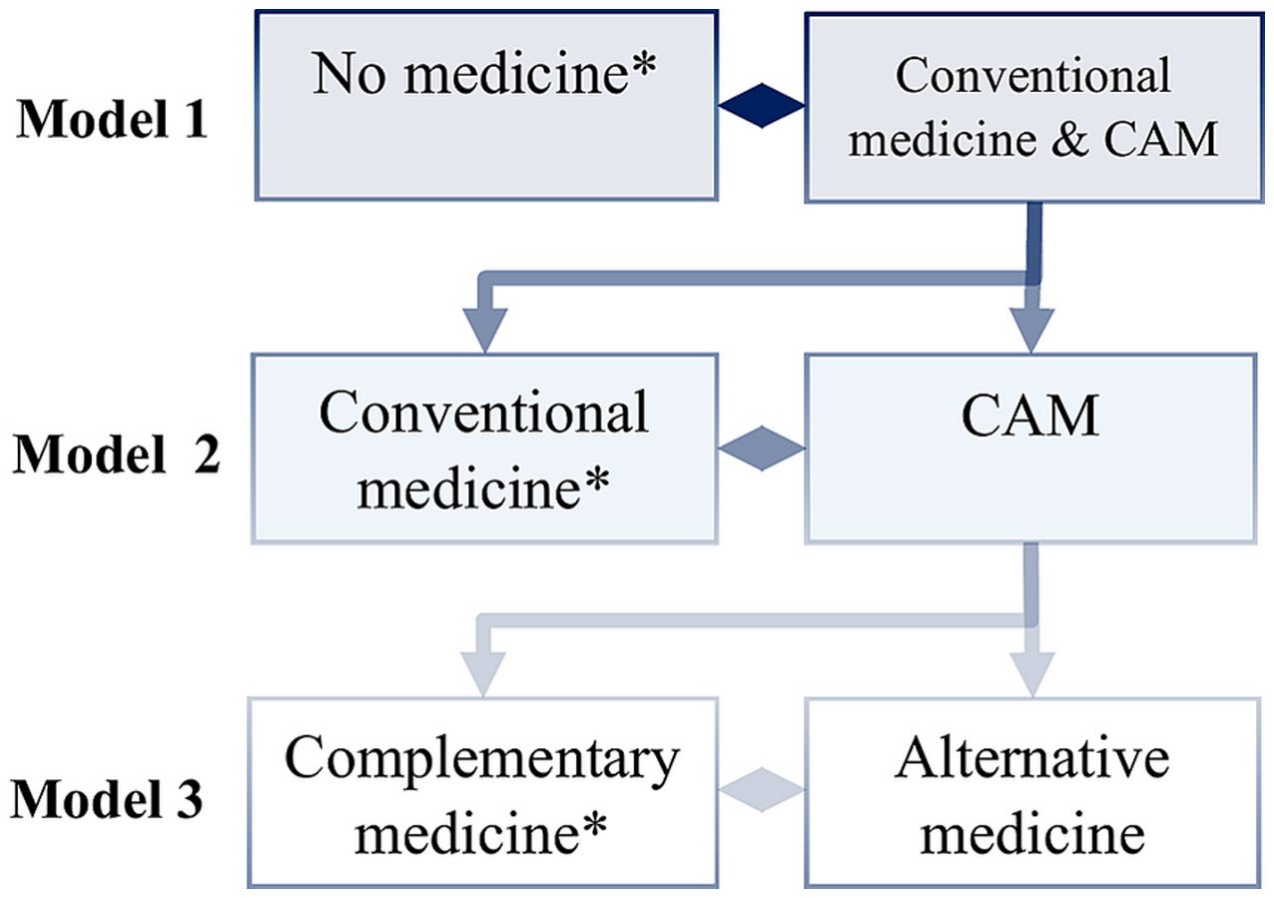
Model structure nested multiple logistic regression. *reference category; CAM = complementary & alternative medicine; own illustration.

Three separate models are estimated. The first model compares non-users with individuals who use both conventional medicine and CAM. The second model contrasts users of conventional medicine with those who use both forms of CAM. The third model compares users of complementary medicine with users of alternative medicine. The third model is particularly important because it allows us to identify which variables not only positively influence CAM use in general, but also specifically its use as an alternative. This stepwise approach enables the inclusion of all respondents in the analysis and allows for a detailed examination of both the general motivation to use CAM and the differences between types of CAM usage. For an overview of the model structure, see Figure 1.

Odds ratios (OR) are reported for all models, and potential multicollinearity is assessed using variance inflation factor (VIF) coefficients. The overall highest VIF coefficient observed is 3.74 and therefore unproblematic (Kim, 2019). The data set is processed and analyzed using R (especially the R package nestedLogit). For the country variable the United States of America is used as a reference.^2^

## 4 Results

**Figure 2:**
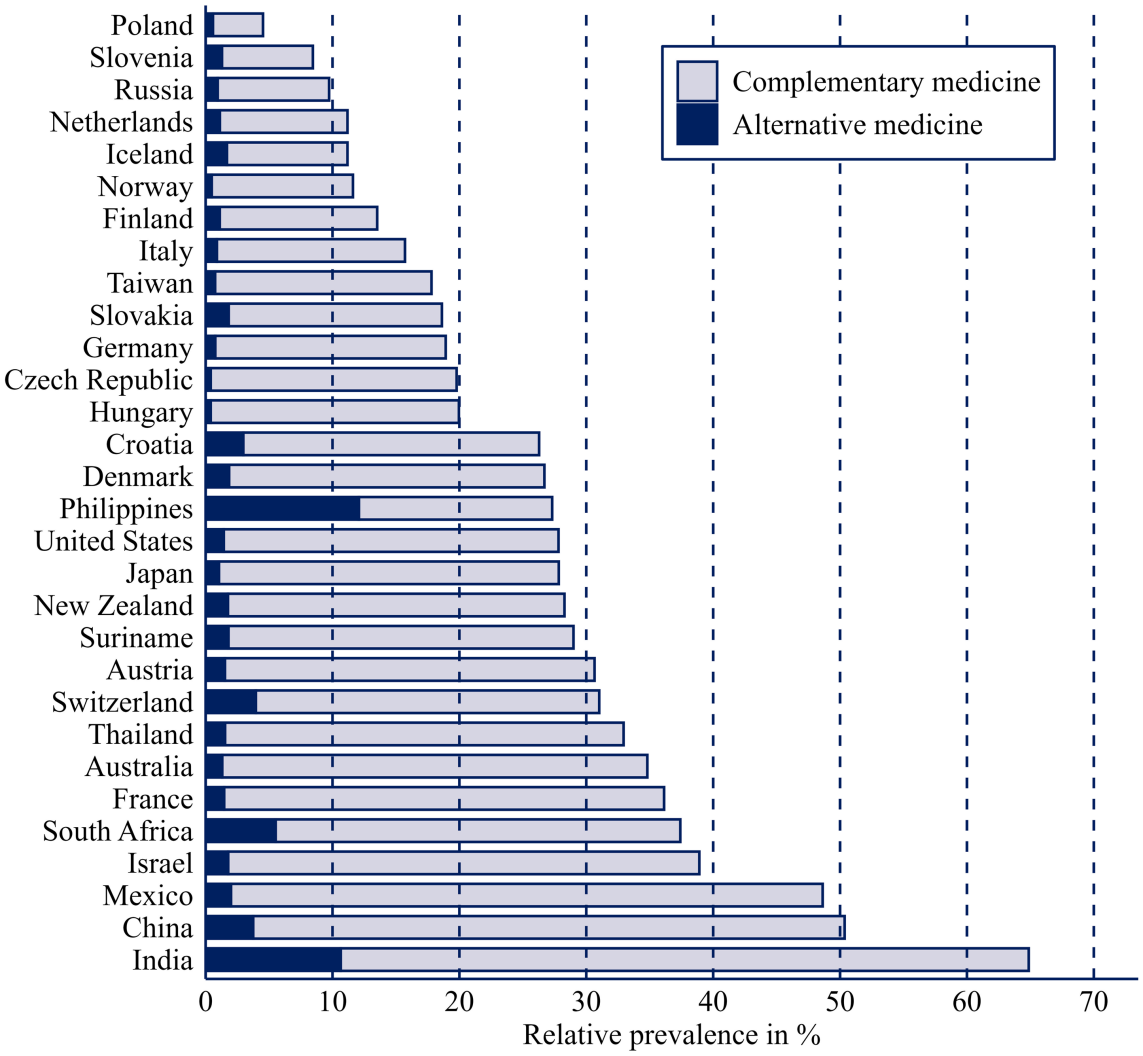
Stacked bar plot showing prevalence of complementary medicine and alternative medicine by country.

Overall, 25.10% of respondents reported using complementary medicine. In contrast, only 2.69% reported using alternative medicine (see Table 3). The top three countries reporting the highest use of complementary medicine are India (54.22%), Mexico (46.63%), and China (46.61%). For alternative medicine usage, the leading countries are the Philippines (12.08%), India (10.65%), and South Africa (5.52%). When examining the ratio of complementary to alternative medicine usage, the Philippines leads substantially with 44.23%, followed by India (16.42%) and Slovenia (15.27%). Overall, these findings highlight substantial variation in the reported use of complementary and alternative medicine worldwide (see Figure 2).

**Table 3:** Prevalence of medical usage.

Variable value	Absolute prevalence	Relative prevalence
No usage	9,125	21.00
Conventional medicine	22,226	51.20
Complementary medicine	10,896	25.10
Alternative medicine	1,167	2.69

The results of the nested logistic regression are presented in Table 4. For a clearer table, and because it is not our focus to explain the usage of the overall medical procedure, model 1 is not included in the table. Results for model 1 can be found in Supplementary Table 5. Affiliation with a religious community is statistically significant in the case of CAM usage (OR_Model2_ = 0.91, *p* ≤ 0.05). In contrast, the frequency of attending religious events is positively and significantly associated with the use of CAM services. Respondents who are satisfied with the healthcare system of their country are more likely to visit a doctor (OR_Model1_ = 1.12, *p* ≤ 0.01) and less likely to use one form of CAM (OR_Model2_ = 0.78, *p* ≤ 0.001). Respondents also refrain from alternative medicine usage when satisfied with the healthcare system (OR_Model3_ = 0.72, *p* ≤ 0.001).

**Table 4:** Nested (multiple) logistic regression on usage of complementary medicine and alternative medicine.

	Model 2 CAM vs. Conventional Medicine (Ref)			Model 3 Alternative Medicine vs. Complementary Medicine (Ref)		
	OR	SE	95%-CI	OR	SE	95%-CI
Variables						
No confession	*Ref.*	*Ref.*	*Ref.*	*Ref.*	*Ref.*	*Ref.*
Confession	0.91*	0.03	[0.85; 0.98]	1.00	0.11	[0.81; 1.24]
Frequency of attending religious events						
Never	*Ref.*	*Ref.*	*Ref.*	*Ref.*	*Ref.*	*Ref.*
Less frequently than once a year	1.22***	0.05	[1.12; 1.33]	1.14	0.15	[0.89; 1.47]
At least one time a year	1.35***	0.05	[1.25; 1.46]	0.83	0.10	[0.66; 1.05]
At least one time a month	1.35***	0.07	[1.22; 1.5]	1.13	0.15	[0.87; 1.48]
At least one time a week	1.28***	0.07	[1.16; 1.42]	0.98	0.14	[0.74; 1.28]
Satisfaction healthcare system						
Neither satisfied nor dissatisfied	*Ref.*	*Ref.*	*Ref.*	*Ref.*	*Ref.*	*Ref.*
Satisfied	0.78***	0.03	[0.72; 0.83]	0.72***	0.06	[0.6; 0.86]
Dissatisfied	0.99	0.05	[0.91; 1.09]	1.09	0.12	[0.88; 1.36]
Gender						
Male	*Ref.*	*Ref.*	*Ref.*	*Ref.*	*Ref.*	*Ref.*
Female	1.29***	0.03	[1.23; 1.36]	0.97	0.07	[0.85; 1.11]
Age						
Metric	0.99***	0.00	[0.99; 0.99]	0.99***	0.00	[0.99; 1]
Education						
No formal education & primary education	*Ref.*	*Ref.*	*Ref.*	*Ref.*	*Ref.*	*Ref.*
Secondary education	0.98	0.05	[0.89; 1.08]	0.78*	0.08	[0.63; 0.95]
Tertiary education	0.95	0.05	[0.86; 1.05]	0.62***	0.07	[0.49; 0.77]
Subjective health status						
Bad & fair	*Ref.*	*Ref.*	*Ref.*	*Ref.*	*Ref.*	*Ref.*
Good	0.92**	0.03	[0.86; 0.98]	1.36**	0.13	[1.13; 1.65]
Very good & excellent	0.91**	0.03	[0.85; 0.97]	2.02***	0.19	[1.68; 2.42]
Country						
United States	*Ref.*	*Ref.*	*Ref.*	*Ref.*	*Ref.*	*Ref.*
Australia	1.49***	0.16	[1.21; 1.83]	0.91	0.36	[0.42; 2]
Austria	1.12	0.11	[0.92; 1.35]	1.05	0.37	[0.53; 2.1]
China	6.01***	0.56	[5; 7.22]	1.63	0.48	[0.91; 2.92]
Croatia	1.13	0.12	[0.92; 1.39]	1.97*	0.65	[1.03; 3.78]
Czech Republic	0.62***	0.07	[0.5; 0.77]	0.40	0.21	[0.14; 1.13]
Denmark	1.18	0.12	[0.97; 1.43]	1.86	0.63	[0.96; 3.6]
Finland	0.49***	0.06	[0.38; 0.62]	1.92	0.80	[0.85; 4.34]
France	1.64***	0.15	[1.37; 1.98]	0.98	0.34	[0.5; 1.95]
Germany	0.59***	0.06	[0.49; 0.72]	0.84	0.34	[0.39; 1.85]
Hungary	0.81	0.09	[0.65; 1.01]	0.34	0.20	[0.11; 1.05]
Iceland	0.42***	0.06	[0.32; 0.54]	3.41**	1.33	[1.59; 7.32]
India	5.00***	0.49	[4.12; 6.06]	2.83***	0.81	[1.62; 4.97]
Israel	1.51***	0.15	[1.24; 1.83]	0.80	0.28	[0.4; 1.6]
Italy	0.43***	0.05	[0.34; 0.54]	1.15	0.50	[0.49; 2.71]
Japan	0.96	0.10	[0.79; 1.17]	1.00	0.39	[0.47; 2.14]
Mexico	2.01***	0.21	[1.64; 2.47]	0.77	0.28	[0.38; 1.59]
Netherlands	0.43***	0.05	[0.33; 0.54]	2.49*	1.02	[1.12; 5.55]
New Zealand	1.13	0.12	[0.92; 1.39]	1.17	0.44	[0.55; 2.45]
Norway	0.36***	0.04	[0.28; 0.45]	0.70	0.37	[0.25; 1.99]
Philippines	3.23***	0.34	[2.62; 3.98]	13.02***	3.75	[7.41; 22.88]
Poland	0.13***	0.02	[0.09; 0.18]	2.48	1.29	[0.9; 6.85]
Russia	0.33***	0.04	[0.26; 0.41]	2.28*	0.88	[1.07; 4.85]
Slovakia	0.57***	0.07	[0.46; 0.72]	1.36	0.53	[0.64; 2.92]
Slovenia	0.35***	0.05	[0.26; 0.46]	3.54**	1.44	[1.59; 7.88]
South Africa	2.18***	0.19	[1.84; 2.6]	2.62***	0.75	[1.5; 4.58]
Suriname	0.78*	0.08	[0.63; 0.95]	1.19	0.43	[0.58; 2.4]
Switzerland	1.46***	0.12	[1.23; 1.72]	2.96***	0.85	[1.69; 5.18]
Taiwan	0.71***	0.07	[0.58; 0.86]	0.98	0.39	[0.45; 2.16]
Thailand	1.34**	0.13	[1.1; 1.62]	0.75	0.28	[0.36; 1.56]
*n*			31,673			11,048
logLik			−18,400.57			−3,158.24
McFadden’s Pseudo-R^2^			11.63			11.42

CAM = complementary and alternative medicine; empirical significance level (z-test, two-sided): ***p ≤ 0.001, **p ≤ 0.01, *p ≤ 0.05.

Country-level differences are substantial. The strongest positive associations with CAM usage are found in China (OR_Model2_ = 6.01, *p* ≤ 0.001), India (OR_Model2_ = 5.00, *p* ≤ 0.001), and the Philippines (OR_Model2_ = 3.23, *p* ≤ 0.001). For alternative medicine, the largest effects are observed in the Philippines (OR_Model3_ = 13.02, *p* ≤ 0.001), Slovenia (OR_Model3_ = 3.54, *p* ≤ 0.001), and Iceland (OR_Model3_ = 3.41, *p* ≤ 0.001).

The control variables show several notable associations. Women are more likely than men to use either conventional medicine or a form of CAM (OR_Model1_ = 1.51, *p* ≤ 0.001). They are also significantly more likely to use one form of CAM instead of solely relying on doctors (OR_Model2_ = 1.29, *p* ≤ 0.001). However, this effect does not hold for the use of alternative medicine (OR_Model3_ = 0.97, *p* > 0.05). Regarding education, tertiary education significantly increases the likelihood of using either conventional medicine or CAM (OR_Model1_ = 1.30, *p* ≤ 0.001). However, education shows no significant effect when comparing both CAM forms to conventional medicine. This changes when looking specifically at alternative medicine use: higher education is associated with a significantly lower likelihood of using alternative medicine (OR_Model3_: secondary = 0.78, *p* ≤ 0.05; OR_Model3_: tertiary = 0.62, *p* ≤ 0.001). This suggests a linear trend, with decreasing odds of alternative medicine use and increasing odds of complementary medicine as the level of education increases. Age is positively associated with not being in the non-user group (OR_Model1_ = 1.01, *p* ≤ 0.001). In contrast, age shows a significant negative association with CAM usage (OR_Model2_ = 0.99, *p* ≤ 0.001; OR_Model3_ = 0.99, *p* ≤ 0.001). Self-rated health has a consistent negative effect on the use of conventional or CAM services, and also on the use of both CAM forms compared to conventional medicine. All these effects are highly significant. In contrast, the relationship with alternative medicine is positive and appears to increase linearly. Conversely, individuals with poorer health are more likely to use conventional medicine, either exclusively or in combination with complementary medicine. Self-rated health has a consistent negative effect on the use of conventional or CAM services, and also on the use of both CAM forms compared to conventional medicine. All these effects are highly significant. In contrast, the relationship with alternative medicine is positive and appears to increase linearly. Conversely, individuals with poorer health are more likely to use conventional medicine, either exclusively or in combination with complementary medicine.

## 5 Discussion

The aim of this study is to differentiate between complementary and alternative medicine usage, and to investigate the extent to which religion and other sociodemographic factors predict these two forms of engagement with non-conventional medicine. By using the large, cross-national dataset from the ISSP Research Group (2024) and applying a nested logistic regression model, the analysis confirms both the empirical relevance and conceptual necessity of distinguishing between complementary and alternative usage patterns.

The results indicate that complementary medicine is far more prevalent than alternative medicine, confirming the first assumption and aligning with prior findings suggesting that complete substitution of conventional medicine is relatively rare (Astin, 1998; Berna et al., 2019). This difference in prevalence underscores the importance of avoiding a homogenized treatment of CAM in empirical research. Instead, separating these categories allows for more precise theorizing and better policy responses, especially in public health settings where the consequences of substituting conventional medicine can be severe (Horneber et al., 2012).

Religion emerges as a more complex and ambivalent predictor. Religious affiliation alone shows no stable effect and even turns slightly negative in model 2, which suggests that formal membership is not a strong driver of CAM use. In contrast, religious practice (measured by attendance) shows a clearer pattern, but only in certain comparisons. Individuals who attend services more often are more likely to use medicine in general and especially to combine conventional medicine with CAM. However, this association weakens when complementary and alternative use are distinguished, as frequent attendance is not linked to the replacement of conventional medicine with alternative approaches. The partial contradiction between religious affiliation and religious practice may be explained by the distinction between organized belief systems and lived religious practices. Individuals who attend services regularly may be more involved in communities that encourage holistic or integrative approaches to health, whereas affiliation alone may not necessarily translate into active spiritual health-seeking (Koenig, 2012; Oman, 2018). Taken together, the results suggest that religious practice is linked mainly to complementary use, not to alternative use. These findings offer only partial support for Hypothesis 1 and highlight the need to differentiate religious variables in future research. These results are in line with international evidence showing that religious groups differ not only in whether they use CAM, but in how they frame it. Several studies argue that CAM practices blend easily with holistic, spiritually oriented worldviews, but are less compatible with traditions that emphasize doctrinal boundaries or scepticism toward “non-traditional” healing (Agarwal, 2018; Hoosen et al., 2021; Soto-Espinosa and Koss-Chioino, 2017; Testerman et al., 2004). This may help explain why religious practice predicts complementary but not alternative use: individuals embedded in religious communities might selectively adopt CAM practices that are perceived as harmonious with spiritual well-being, rather than those that position themselves as substitutes for biomedicine.

Satisfaction with the healthcare system shows a consistent association with patterns of medicine use. Individuals who report being satisfied are more likely to use CAM in addition to conventional medicine, compared to not using any treatment at all. However, satisfaction reduces the odds of turning to CAM as a substitute for conventional care, both when comparing CAM to conventional medicine and when distinguishing complementary from alternative use. By contrast, dissatisfaction with the healthcare system does not display a significant relationship. These findings suggest that CAM is not necessarily a response to low satisfaction, but rather that satisfied patients integrate CAM as a complement, while remaining committed to conventional medicine.

The analysis also reveals a clear variation in CAM usage between countries. Countries such as the Philippines, India, China and South Africa display much higher rates of alternative medicine use. These patterns likely reflect both cultural traditions and structural aspects of healthcare systems. In some regions, traditional medicine is deeply integrated into daily life and may be more accessible or affordable than conventional medicine. Limited access to conventional medicine due to financial constraints or underfunded health infrastructures may also drive people to rely on traditional or alternative options. In contrast, in countries with robust healthcare systems, CAM may serve more as a supplement for lifestyle or well-being purposes rather than an alternative for medical treatment. Some results, such as the very high odds ratios observed for the Philippines (OR = 13.02 in Model 3) need to be interpreted with caution. The large standard errors suggest that these estimates may be influenced by small group sizes or model instability rather than reflecting precise differences in CAM use. These country-specific patterns imply different public health responses. In countries with high alternative medicine use such as the Philippines, India, or South Africa, policy efforts should focus on improving access to affordable conventional care and promoting safe integration of traditional practitioners into formal healthcare systems. Clear communication about the risks of substituting conventional treatments is essential, particularly for vulnerable populations with limited healthcare access. In contrast, in high-income countries like Germany, France, or Switzerland, where complementary rather than alternative use predominates, policymakers could support evidence-based integration of selected CAM practices into preventive and rehabilitative care. Public information campaigns should emphasize informed decision-making and encourage transparent dialogue between providers of conventional medicine and alternative medicine to ensure patient safety and coordinated treatment.

Further insights emerge when examining the control variables. The gender effect is significant only for complementary use and not for alternative use, suggesting that women may turn to CAM primarily to supplement rather than replace conventional medicine. This aligns with prior research suggesting that women are generally more proactive in health maintenance and are more open to integrating multiple approaches to well-being (Zhang et al., 2015). Higher education is strongly negatively associated with alternative medicine use. This supports the notion that more educated individuals may be better equipped to critically assess health information and are less likely to rely exclusively on non-evidence-based treatments (Čavojová and Ersoy, 2020; Cutler and Lleras-Muney, 2006). These abilities align with broader concepts of digital and critical health literacy, which refer to individuals’ capacity to evaluate the trustworthiness of health information; especially in digital environments where CAM-related claims are widespread (Chinn and McCarthy, 2013; Sørensen et al., 2021). Age also demonstrates a distinct association with CAM usage. While older age is slightly associated with increased medicine usage overall, the odds of using CAM complementary as well as an alternative declines with age. This result is consistent with studies that show younger people may be more inclined toward holistic lifestyles and less attached to conventional care (Fjær et al., 2020; Wemrell et al., 2017). Younger age groups also tend to engage more with digital health information environments, which may expose them more strongly to CAM-related content and contribute to the higher uptake of complementary or alternative practices.

Some limitations should be noted. First, the operationalization of CAM is based on reported visits to healthcare practitioners. The question of whether a person visited an alternative health care practitioner does not allow for a differentiated look into the various forms, methods and therapies of CAM. It is also important to note that depending on the country and language the item is not exactly the same. Words like alternative, traditional and folk health care practitioners are used, further complicating estimation. This likely results in a conservative estimate of CAM usage. Therefore, CAM usage is more likely to be underestimated than overestimated. Second, although religious confession and attendance are included, measures of spirituality, religious beliefs, or non-institutional forms of religiosity were not available. These could provide further explanatory power in future analyses. Consequently, our analysis mainly captures the institutional and behavioral dimensions of religion, such as formal affiliation and public participation, but not the personal strength of belief or the ways in which individuals practice their faith privately. Therefore, the observed associations should be interpreted with this limitation in mind: they primarily reflect the effects of outward religious involvement, rather than inner conviction, belief intensity, or private spiritual expression. Thirdly, our set of explanatory variables is narrow. While this approach allows for clear analysis, it inevitably overlooks other potential determinants of CAM use. Numerous factors may also play a role in shaping CAM utilization. Future research is therefore encouraged to expand upon our findings by incorporating a broader range of explanatory variables in order to achieve a more comprehensive understanding of the drivers of CAM use.

This study provides important evidence on the patterns and determinants of CAM use in a large international sample. By differentiating between complementary and alternative practices, the analysis reveals that complementary use is much more prevalent than exclusive alternative use, and that religiosity, education, age, and gender are key factors shaping these behaviors. These findings advance the understanding of CAM utilization and highlight the need to consider sociodemographic diversity in both research and policy. As healthcare becomes more diverse, understanding why people turn to CAM can help create strategies that improve patient-centered care and address differences in health outcomes. Additionally, gaining a better awareness of how religion influences health behaviors can enable healthcare providers to connect more effectively with patients from various backgrounds, promoting culturally sensitive care. These findings carry important implications. Theoretically, they highlight the value of distinguishing between complementary and alternative health behaviors in order to uncover different motivational structures, risk profiles, and social predictors. From a policy perspective, public health campaigns must be tailored to specific forms of CAM engagement. Interventions that seek to minimize the risks of medical non-compliance should target alternative users in particular. At the same time, complementary use, when integrated safely, could be leveraged to enhance patient satisfaction and overall health outcomes, especially in preventive care and chronic disease management. Future research should consider comparative analysis across countries and health systems which could also provide valuable insights into how policy environments and access barriers influence CAM utilization on a population level. Additionally, future studies might explore alternative operationalizations of CAM use; for example, by capturing private or informal use, as well as attitudes and motivations toward CAM. This would allow researchers to go beyond reported visits to healthcare practitioners and gain a more comprehensive understanding of CAM engagement.

## Data Availability

Publicly available datasets were analyzed in this study. This data can be found here: data and replication code for the analysis can be found at https://osf.io/52rmz/?view_only=0864343f132342f6a2bee1c1c51ead20.

## Footnotes

1 For more information about the ISSP Research Group (2024) see https://www.gesis.org/en/issp/data-and-documentation/health-and-health-care/2021.

2 For the reference country, we identify the nation whose complementary and alternative medicine (CAM) usage are closest to the overall mean (M = 27.79%). Because Japan and the United States of America (USA) both have a mean of 27.80%, we use the ratio of alternative to complementary medicine at the country level (M = 9.46%) as a secondary variable. The USA is selected as the reference category, as its value (5.14%) is closer to the mean than that of Japan (3.77%). The aim of this procedure is to identify a country positioned near the center of the distribution.
