# Supplementary material for: Distinguishing complementary and alternative medicine: the role of religion, healthcare system satisfaction and country context
Source: Front Sociol. 2026 Jan 16;10:1702000. doi: 10.3389/fsoc.2025.1702000 (PMC12857057; doi:10.3389/fsoc.2025.1702000)
Supplement: Supplementary file 1 [file Supplementary_file_1.docx]

Supplementary Material

# Supplementary Tables

Table 1: Sample by country of residence

| **Country** | **n** | **%** |
| --- | --- | --- |
| Australia | 1,050 | 2.4 |
| Austria | 1,546 | 3.5 |
| China | 2,689 | 6.0 |
| Croatia | 1,101 | 2.5 |
| Czech Republic | 1,262 | 2.8 |
| Denmark | 1,672 | 3.8 |
| Finland | 1,002 | 2.2 |
| France | 1,584 | 3.6 |
| Germany | 1,744 | 3.9 |
| Hungary | 1,008 | 2.3 |
| Iceland | 1,086 | 2.4 |
| India | 1,683 | 3.8 |
| Israel | 1,187 | 2.7 |
| Italy | 1,138 | 2.6 |
| Japan | 1,453 | 3.3 |
| Mexico | 1,001 | 2.2 |
| Netherlands | 1,269 | 2.8 |
| New Zealand | 1,135 | 2.5 |
| Norway | 1,518 | 3.4 |
| Philippines | 1,800 | 4.0 |
| Poland | 1,098 | 2.5 |
| Russia | 1,597 | 3.6 |
| Slovakia | 1,013 | 2.3 |
| Slovenia | 1,020 | 2.3 |
| South Africa | 2,829 | 6.4 |
| Suriname | 1,468 | 3.3 |
| Switzerland | 3,349 | 7.5 |
| Taiwan | 1,604 | 3.6 |
| Thailand | 1,497 | 3.4 |
| United States of America | 1,050 | 2.6 |
| Sum | 44,549 | ≈ 100.0 |

Percentage does not sum to exactly 100 due to rounding errors

Table 2.1: Descriptive sample analysis – categorical variables

| **Variable** | **n** | **%** |
| --- | --- | --- |
| *CAM usage*  No Medicine  Conventional medicine  Complementary medicine  Alternative medicine | 9,125  22,226  10,896  1,167 | 21.02  51.20  25.10  2.7 |
| *Confession dummy*  Confession  No Confession | 30,085  13,457 | 69.1  30.9 |
| *Frequency of attending religious events*  Never  Less frequently than once a year  At least once a year  At least one time a month  At least one time a week | 15,339  6,010  11,698  5,038  5,055 | 35.6  13.9  27.1  11.7  11.7 |
| *Satisfaction healthcare system*  Satisfied  Neither satisfied nor dissatisfied  Dissatisfied | 7,185  29,898  6,596 | 16.4  68.4  15.1 |
| *Gender* Male  Female | 20,545  23,928 | 46.20  53.80 |
| *Education*  No formal education & primary education  Secondary education  Tertiary education | 4,486  23,122  16,261 | 10,23  52,71  37,07 |
| *Subjective health status*  Bad & fair  Good  Very good & excellent | 11,991  16,715  15,394 | 27.2  37.9  34.9 |

Percentages do not sum to exactly 100 due to rounding errors

Table 2.2: Descriptive sample analysis – numerical variables

| **Variable** | **Min.** | **1^st^ Quartil** | **Median** | **3^rd^ Quartil** | **Max.** | **Mean** | **Standard Deviation** |
| --- | --- | --- | --- | --- | --- | --- | --- |
| *Age* | 16 | 35 | 49 | 63 | 111 | 49.46 | 17.46 |

Table 3: Variable coding

| **Variable** | **Variable name** | **Question text and original labelling** | **Own label (original label)** |
| --- | --- | --- | --- |
| CAM usage | v38 & v39 | During the past 12 months, how often did you visit or were visited by …  v38: ...a doctor?  v39: ...an [alternative/ traditional/ folk] health care practitioner?  1=Never  2=Seldom  3=Sometimes  4=Often  5=Very often | 1=No medicine (v38=1 & v39=1)  2=Conventional medicine (v38=2,3,4,5 & v39=1)  3=Complementary medicine (v38=2,3,4,5 & v39=2,3,4,5)  4= Alternative medicine (v38=1 & v39=2,3,4,5) |
| Confession group | religgrp | *The question texts vary by country (for original question texts see ISSP2021 variables from AT_RELIG to ZA_RELIG).*  0=No religion  1=Catholic  2=Protestant  3=Orthodox  4=Other Christian  5=Jewish  6=Islamic  7=Buddhist  8=Hindu  9=Other Asian Religions  10=Other Religions | 1=No confession (0)  2=Confession (1 to 10) |
| Frequency of attending religious events | attend | Apart from such special occasions as weddings, funerals, etc., how often do you attend religious services?  1=Several times a week or more often  2=Once a week  3=2 or 3 times a month  4=Once a month  5=Several times a year  6=Once a year; CH, HU, US: Once or twice a year  7=Less frequently than once a year  8=Never | 1=Never (8)  2=Less frequently than once a year (7)  3=At least once a year (5,6)  4=At least one time a month (3,4)  5=At least one time a week(1,2) |
| Satisfaction healthcare system | v44 | In general, how satisfied or dissatisfied are you with the health care system in [country]?  1=Completely satisfied  2=Very satisfied  3=Fairly satisfied  4=Neither satisfied nor dissatisfied  5=Fairly dissatisfied  6=Very dissatisfied  7=Completely dissatisfied | 1=Neither satisfied nor dissatisfied (4)  2=Satisfied (1,2,3)  3=Dissatisfied (5,6,7) |
| Gender | sex | Are you...?  1=Male  2=Female | 1=Male (1)  2=Female (2) |
| Age | age | *Age is either derived from birthyear or from the following question.*  What was your age at your last birthday, in full years? | Open question |
| Education | edulevel | *Education is based on the following question, with an additional simplification according to ISCED 2011 scale*.  What is the highest level of education you have ever completed?  0=No formal education  1=Primary education  2=Lower secondary  3=Upper secondary  4=Post secondary, non-tertiary  5=Short-cycle tertiary  6=Lower level tertiary, BA  7=Upper level tertiary, MA  8=PhD, Post Tertiary Specialization | 1=No formal education & primary education (0,1)  2=Secondary education (2,3,4)  3=Tertiary education (5,6,7,8) |
| Subjective Health | v51 | In general, would you say your health is …  1=Excellent  2=Very good  3=Good  4=Fair  5=Poor | 1=Bad & Fair (4&5)  2=Good (3)  3=Very good & excellent (1&2) |
| Country | country | *The country of residence is not directly asked.*  36=Australia  40=Austria  156=China  158=Taiwan  191=Croatia  203=Czech Republic  208=Denmark  246=Finland  250=France  276=Germany  348=Hungary  352=Iceland  356=India  376=Israel  380=Italy  392=Japan  484=Mexico  528=Netherlands  554=New Zealand  578=Norway  608=Philippines  616=Poland  643=Russia  703=Slovakia  705=Slovenia  710=South Africa  740=Suriname  756=Switzerland  764=Thailand  840=United States | 1=United States  2=Australia  3=Austria  4=China  5=Croatia  6=Czech Republic  7=Denmark  8=Finland  9=France  10=Germany  11=Hungary  12=Iceland  13=India  14=Israel  15=Italy  16=Japan  17=Mexico  18=Netherlands  19=New Zealand  20=Norway  21=Philippines  22=Poland  23=Russia  24=Slovakia  25=Slovenia  26=South Africa  27=Suriname  28=Switzerland  29=Taiwan  30=Thailand |

Table 4: Missing value rates

|  | **n** | **%** |
| --- | --- | --- |
| CAM usage | 1,135 | 2.55% |
| Confession | 1,007 | 2.26% |
| Frequency of attending religious events | 1,409 | 3.16% |
| Satisfaction healthcare system | 870 | 1.95% |
| Gender | 76 | 0.17% |
| Age | 293 | 0.66% |
| Education | 680 | 1.53% |
| Subjective health status | 449 | 1.01% |
| Country | 0 | 0.00% |
| Sum/Mean | 6,926 | 1.55% |

Table 5: Nested (multiple) logistic regression on usage of complementary medicine and alternative medicine

|  | **Model 1**  **No Medicine vs. Conventional Medicine and CAM (Ref)** | | |
| --- | --- | --- | --- |
|  | **OR** | **SE** | **95%-CI** |
| **Confession** |  |  |  |
| No confession | *Ref.* | *Ref.* | *Ref.* |
| Confession | 0.94 | 0.04 | [0.88; 1.02] |
| **Frequency of attending religious events** |  |  |  |
| Never | *Ref.* | *Ref.* | *Ref.* |
| Less frequently than once a year | 1.12* | 0.05 | [1.03; 1.22] |
| At least one time a year | 1.2*** | 0.05 | [1.11; 1.3] |
| At least one time a month | 1.31*** | 0.07 | [1.18; 1.45] |
| At least one time a week | 1.44*** | 0.08 | [1.3; 1.61] |
| **Satisfaction healthcare system** |  |  |  |
| Neither satisfied nor dissatisfied | *Ref.* | *Ref.* | *Ref.* |
| Satisfied | 1.12** | 0.04 | [1.04; 1.2] |
| Dissatisfied | 0.94 | 0.04 | [0.86; 1.03] |
| **Gender** |  |  |  |
| Male | *Ref.* | *Ref.* | *Ref.* |
| Female | 1.51*** | 0.04 | [1.43; 1.59] |
| **Age** |  |  |  |
| metric | 1.01*** | 0.00 | [1.01; 1.01] |
| **Education** |  |  |  |
| No formal education & primary education | *Ref.* | *Ref.* | *Ref.* |
| Secondary education | 1.05 | 0.05 | [0.96; 1.16] |
| Tertiary education | 1.3*** | 0.07 | [1.17; 1.44] |
| **Subjective health status** |  |  |  |
| Bad & fair | *Ref.* | *Ref.* | *Ref.* |
| Good | 0.63*** | 0.02 | [0.58; 0.67] |
| Very good & excellent | 0.41*** | 0.02 | [0.38; 0.44] |
| **Country** |  |  |  |
| United States | *Ref.* | *Ref.* | *Ref.* |
| Australia | 2.74*** | 0.48 | [1.94; 3.87] |
| Austria | 2.3*** | 0.33 | [1.74; 3.03] |
| China | 0.42*** | 0.04 | [0.34; 0.51] |
| Croatia | 0.41*** | 0.05 | [0.33; 0.51] |
| Czech Republic | 1.52** | 0.20 | [1.18; 1.96] |
| Denmark | 0.63*** | 0.07 | [0.5; 0.78] |
| Finland | 0.56*** | 0.07 | [0.44; 0.71] |
| France | 1.63*** | 0.21 | [1.26; 2.1] |
| Germany | 2.14*** | 0.29 | [1.64; 2.78] |
| Hungary | 0.62*** | 0.07 | [0.49; 0.78] |
| Iceland | 0.3*** | 0.03 | [0.24; 0.38] |
| India | 1.95*** | 0.25 | [1.52; 2.5] |
| Israel | 2.53*** | 0.38 | [1.89; 3.39] |
| Italy | 1.21 | 0.16 | [0.93; 1.57] |
| Japan | 0.88 | 0.11 | [0.69; 1.12] |
| Mexico | 2.56*** | 0.44 | [1.82; 3.59] |
| Netherlands | 0.37*** | 0.04 | [0.29; 0.46] |
| New Zealand | 1.34* | 0.18 | [1.02; 1.75] |
| Norway | 0.93 | 0.11 | [0.74; 1.18] |
| Philippines | 0.13*** | 0.01 | [0.1; 0.15] |
| Poland | 0.37*** | 0.04 | [0.29; 0.46] |
| Russia | 0.29*** | 0.03 | [0.23; 0.36] |
| Slovakia | 0.84 | 0.11 | [0.66; 1.07] |
| Slovenia | 0.23*** | 0.03 | [0.18; 0.29] |
| South Africa | 0.41*** | 0.04 | [0.34; 0.5] |
| Suriname | 0.87 | 0.11 | [0.68; 1.11] |
| Switzerland | 0.85 | 0.09 | [0.69; 1.04] |
| Taiwan | 0.45*** | 0.05 | [0.36; 0.55] |
| Thailand | 0.6*** | 0.07 | [0.48; 0.75] |
| n |  |  | 40,182 |
| logLik |  |  | -18,352.53 |
| McFadden’s Pseudo-R^2^ |  |  | 11.53 |

CAM = complementary and alternative medicine; empirical significance level (z-test, two-sided): ***p ≤ 0.001, **p ≤ 0.01, *p ≤ 0.05
